# Lycopene and Beta-Carotene Induce Growth Inhibition and Proapoptotic Effects on ACTH-Secreting Pituitary Adenoma Cells

**DOI:** 10.1371/journal.pone.0062773

**Published:** 2013-05-07

**Authors:** Natália F. Haddad, Anderson J. Teodoro, Felipe Leite de Oliveira, Nathália Soares, Rômulo Medina de Mattos, Fábio Hecht, Rômulo Sperduto Dezonne, Leandro Vairo, Regina Coeli dos Santos Goldenberg, Flávia Carvalho Alcântara Gomes, Denise Pires de Carvalho, Mônica R. Gadelha, Luiz Eurico Nasciutti, Leandro Miranda-Alves

**Affiliations:** 1 Instituto de Ciências Biomédicas, Universidade Federal do Rio de Janeiro, Brazil; 2 Serviço de Endocrinologia, Hospital Universitário Clementino Fraga Filho, Universidade Federal do Rio de Janeiro, Brazil; 3 Instituto de Biofísica Carlos Chagas Filho, Universidade Federal do Rio de Janeiro, Brazil; 4 Programa de Nutrição e Alimentos, Universidade do Estado do Rio de Janeiro, Brazil; Harvard Medical School, United States of America

## Abstract

Pituitary adenomas comprise approximately 10–15% of intracranial tumors and result in morbidity associated with altered hormonal patterns, therapy and compression of adjacent sella turcica structures. The use of functional foods containing carotenoids contributes to reduce the risk of chronic diseases such as cancer and vascular disorders. In this study, we evaluated the influence of different concentrations of beta-carotene and lycopene on cell viability, colony formation, cell cycle, apoptosis, hormone secretion, intercellular communication and expression of connexin 43, Skp2 and p27^kip1^ in ACTH-secreting pituitary adenoma cells, the AtT20 cells, incubated for 48 and 96 h with these carotenoids. We observed a decrease in cell viability caused by the lycopene and beta-carotene treatments; in these conditions, the clonogenic ability of the cells was also significantly decreased. Cell cycle analysis revealed that beta-carotene induced an increase of the cells in S and G2/M phases; furthermore, lycopene increased the proportion of these cells in G0/G1 while decreasing the S and G2/M phases. Also, carotenoids induced apoptosis after 96 h. Lycopene and beta-carotene decreased the secretion of ACTH in AtT20 cells in a dose-dependent manner. Carotenoids blocked the gap junction intercellular communication. In addition, the treatments increased the expression of phosphorylated connexin43. Finally, we also demonstrate decreased expression of S-phase kinase-associated protein 2 (Skp2) and increased expression of p27^kip1^ in carotenoid-treated cells. These results show that lycopene and beta-carotene were able to negatively modulate events related to the malignant phenotype of AtT-20 cells, through a mechanism that could involve changes in the expression of connexin 43, Skp2 and p27^kip1^; and suggest that these compounds might provide a novel pharmacological approach to the treatment of Cushing’s disease.

## Introduction

Dysfunction of the pituitary gland can be caused by a wide variety of diseases such as hypopituitarism and tumors, which may produce major clinical manifestations. Pituitary adenomas are common neoplasms, reported to account for about 10–15% of all intracranial tumors, and are therefore the second most common neoplasm after meningioma. Pituitary adenomas are probably much more common than previously assumed; their prevalence is roughly 1 per 1000 people [1], [2]. These tumors have a monoclonal origin and are classified as endocrine-active or -inactive adenomas, whereas pituitary carcinomas are extremely rare [3].

In general, pituitary tumors are not metastatic. However, they do result in morbidity caused by altered hormonal patterns, therapeutic side effects, and compression of adjacent sella turcica structures [4], [5], [6]. Corticotropinomas are tumors that secrete high ACTH levels, resulting in Cushing’s disease (CD) [4]. It is important to highlight that in ACTH-secreting pituitary tumors, responsible for pituitary-dependent CD, only a small cell population responds to conventional treatment with dopamine agonists or somatostatin analogs, and transsphenoidal surgery remains the primary therapeutic option. These corticotrophic cell adenomas, or corticotropinomas, are responsible for approximately 8% of all clinically recognized pituitary adenomas [7]. So far, the only therapeutic option for adrenal tumor or ectopic ACTH secretion is surgical removal. However, a significant amount of patients cannot be submitted to surgical procedure. Therefore, further studies are necessary either to control tumor development or to provide novel targets for pharmacological therapy. [2], [8].

The recent search for new antitumor drugs has focused mainly on natural compounds obtained from the normal human diet, because these compounds rarely exhibit severe side effects, and yet act efficiently on a wide range of molecular targets involved in tumorigenesis [10], [12]. Several studies have pointed out that the consumption of carotenoids is associated with reduced risk of chronic diseases, including cancer and vascular diseases [12]–[15].

Carotenoids are a family of more than 700 natural lipid-soluble pigments that are produced by higher plants, algae, fungi and bacteria [9]. One promising compound, which is now being tested in clinical studies, is the carotenoid lycopene [10]. Epidemiological studies have suggested that increased consumption of lycopene and beta-carotene is associated with a 30–40% reduction in the risk of prostate cancer [11]. Other studies have demonstrated that lycopene and beta-carotene modulate the cell cycle and induce apoptosis in different tumor lineages. In addition, beta-carotene-rich tomato lycopene beta-cyclase (tlcy-b) inhibits the growth of HT-29 colon adenocarcinoma cancer cells [12]. Tang et al. [13] observed that lycopene inhibits the growth of human colon cancer cells via suppression of the Akt signaling pathway and downstream target molecules, such as cyclin-dependent kinase inhibitor p27^kip1^ and retinoblastoma tumor suppressor protein. Furthermore, previous studies demonstrated that these compounds play a crucial role in the control of intercellular communication through connexin expression modulation,. Consequently the maintenance of homeostasis, morphogenesis, cell differentiation, growth control, apoptosis and hormone secretion in multicellular organisms [14]–[16]. Recent reports have indicated that connexin proteins act in the control of cell growth and death by a mechanism independent of their channel activity [17].

The aim of the present study was to determine the effects of lycopene and beta-carotene on AtT-20 cell viability, clonogenic ability, cell cycle, apoptosis and ACTH secretion. We show that the carotenoids act on ACTH-secreting pituitary adenoma cells by regulating the phosphorylation of connexin 43, and the content of Skp2 and p27^Kip1^.

## Materials and Methods

### Reagents

All-trans lycopene was purchased from Sigma Chemical Company (St. Louis, USA). Water-soluble lycopene and cold-water-soluble beta-carotene were supplied by Roche (Rio de Janeiro, Brazil). For cell culture, Dulbecco's modified culture medium (DMEM) and bovine serum albumin were obtained from Sigma, and fetal bovine serum (FBS) from LABORCLIN (São Paulo, Brazil). Tissue culture flasks and cell scrapers were obtained from Nunc (Roskilde, Denmark). All chemicals were of analytical grade.

### Culture of AtT-20 Cell Line

The mouse corticotroph tumor cell line AtT-20 [18], [19] was kindly provided by Dr Ulrich Renner and Prof. Dr. Günter K. Stalla from the Clinical Neuroendocrinology Group, Max Planck Institute of Psychiatry, Germany. The cells were plated in 25 cm^2^ flasks (TPP Techno Plastic Products, Trasadingen, Switzerland) and routinely maintained in Dulbecco's modified culture medium supplemented with 10% fetal bovine serum (FBS) (LGC Biotechnology, São Paulo, Brazil), 100 U/mL penicillin (LGC Biotechnology, São Paulo, Brazil) and 0.1 mg/mL streptomycin (LGC Biotechnology, São Paulo, Brazil) at 37°C and 5% CO_2_. When the cells reached confluence, they were washed three times with balanced saline solution calcium/magnesium free (BSS-CMF), and a solution of trypsin/EDTA 0.125% (Sigma, St. Louis, USA) was added to detach the cells. The cell suspension was then centrifuged for 5 min at 1340×*g*, and the cells were resuspended in DMEM supplemented with 10% FBS. The number of cells was determined using a Neubauer chamber (New Optik, São Paulo, Brazil).

### Cell Viability

AtT-20 cell viability was determined by MTT assay (Sigma, St. Louis, USA). 1.0×10^4^ cells/well with DMEM were plated in 96-well plates (Corning, New York, USA) at 200 µL/well and then incubated for 3 h according to manufacture procedure. Then, for the treatments, either lycopene or beta-carotene (0.5–40 µM) for 48 h and 96 h, 20 µL/well MTT (5 mg/mL) was added. The medium was then removed after 4 h incubation, and 50 mL of DMSO was added to dissolve the reduced formazan product. Finally, the plate was read in an enzyme-linked immunosorbent microplate reader (Bio-Rad 2550, California, USA) at 570 nm. The cell proliferation inhibition rate (CPIR) was calculated using the following formula: CPIR = (1–mean A value of experimental group/mean A value of control group)×100%. This term (A) corresponding the optical density (OD) average obtained.

### Test of Colony Formation (CFU)

AtT-20 cells were plated at a density of 10^3^ cells/per well in a 6-well plate in DMEM culture medium containing 10% FBS for 48 h. Then, the cells were treated with lycopene or beta-carotene at 5 and 10 µM. The medium was replaced every 5 days. After 21 days, colonies were fixed with 4% paraformaldehyde (Sigma, St. Louis, USA) in PBS containing 4% sucrose (Vetec, Rio de Janeiro, Brazil) for 20 min, and then stained with 0.005% crystal violet (Vetec, Rio de Janeiro, Brazil) overnight at room temperature. The next day, they were washed five times with PBS for 5 min. Colonies containing >50 cells were counted using an Axiovert inverted microscope (Carl Zeiss, Oberkochen, Germany).

### Cell Cycle

After 48 and 96 h of beta-carotene or lycopene treatment (5 and 10 µM), AtT-20 cells were quickly washed with buffered saline (BSS) calcium/magnesium free and were detached with the aid of trypsin at room temperature. After centrifugation, the cells were washed twice with PBS, and then 1×10^6^ cells were resuspended in 1 mL of cold Vindelov’s solution [20], containing 0.1% Triton X-100, 0.1% citrate buffer, 0.1 mg/mL RNase and 50 µg/mL iodide (Sigma, St. Louis, USA). After 15 min, the cell suspension was filtered and analyzed for DNA content by flow cytometry using a FACSCalibur flow cytometer (Becton Dickinson CA, New Jersey, USA). The relative proportions of cells with DNA content indicative of apoptosis (<2 n), G0/G1 diploid (2 n), S (phase >2 n but <4 n), and G2/M phase (4 n) were obtained and analyzed using the CellQuest WinMDI 2.9. The percentage of cell population in a particular phase was estimated by analysis using the Expo32 V1.2 software.

### Apoptosis Assay

AtT-20 cells were treated with lycopene or beta-carotene (5 and 10 µM) for 48 and 96 h. The non-adherent cells were collected, and adherent cells were quickly washed with buffered saline (BBS) calcium/magnesium free and were detached with trypsin/EDTA 0.125% (Sigma, St. Louis, USA) at room temperature. Subsequently, apoptotic and necrotic cells were stained with annexin V-FITC/propidium iodide (PI) (BD Pharmingen, New Jersey, USA) according to the manufacturer’s instructions, quantified by flow cytometer (FACSCalibur, BD Bioscience, New Jersey, USA) and analyzed using two specific programs, Cell Quest and WinMDI 2.9.

### Hormone Quantification

The ACTH secreted by AtT-20 cells treated for 24 h with lycopene or beta-carotene (5 and 10 µM) was measured. The conditioned medium was harvested, and ACTH concentration was determined by using a commercial Immulite 1000 Kit Immunoassay System (Siemens, São Paulo, Brazil) according to the manufacturer’s instructions. The samples were diluted at 1∶100 in DMEM supplemented with 10% FBS. The ACTH values obtained were normalizing using viable cells number determined by MTT assay.

### Enzyme-linked Immunosorbent Assay (ELISA)

A quantitative indirect immunoenzyme assay was performed after the protein levels were measured. Polystyrene microtiter plate wells Maxisorp (Nunc, Roskilde, Denmark) were coated with 50 µL of protein (5 µg/mL in PBS) by passive adsorption overnight at 4°C. The plates were then washed with PBS containing 0.05% Tween 20 and 0.1% BSA (PBS-Tween). Non-specific binding was blocked by incubating the plates for 2 h with 1% BSA in PBS, pH 7.4 at 37°C. After an additional wash with PBS-Tween, the plates were incubated with the primary antibodies: rabbit anti-phospho Cx43 (Sigma, St. Louis, USA) 1∶1000, mouse anti-Cx43 (Invitrogen, USA) 1∶500 and mouse anti-α-tubulin as standard protein (Sigma, St. Louis, USA) 1∶3000 for 24 h at 4°C, followed by incubation with a goat anti-rabbit or mouse IgG peroxidase-linked conjugated antibody (1∶8000, Amersham Biosciences, Buckinghamshire, United Kingdom). The plates were washed with PBS-Tween, and the reaction was developed with the substrate o-phenylenediamine (0.5 mg/mL and 0.005% H_2_O_2_ in 0.01 M sodium citrate buffer, pH 5.6) (Vetec, Rio de Janeiro, Brazil). The reaction was stopped with 0.2 M H_2_SO_4_ (Vetec, Brazil), and the absorbance was measured using an automated reader (BioRad ELISA Reader, Hercules, CA, USA).

### Injection of Iontophoretic Dye

AtT-20 cells treated for 48 h with beta-carotene or lycopene (5 and 10 µM), in a culture plate of 35 mm in diameter, were injected with Lucifer Yellow CH dye (5% in 150 mM in LiCl) using glass microelectrodes with a resistance of 20–50 MΩ) by applying small hyperpolarizing current pulses (9.1 nA, 100 ms), using a WPI amplifier (model 7060). The set microelectrode and Ag/AgCl electrode were coupled to a hydraulic micromanipulator (Nikon, Tokyo, Japan) that enables the microelectrode to access the interior of a single cell. The transfer of dye was observed under an Axiovert 100 inverted microscope (Carl Zeiss, Oberkochen, Germany) equipped with xenon arc illumination. Photomicrographs were taken from random fields using a digital capture system (Image Pro) coupled to the microscope (Carl Zeiss, Oberkochen, Germany). For each experimental condition, 10 injections/plate were made. Data were statistically analyzed with the test of proportions, using a significance level of *p*<0.05.

### Western Blotting for Skp2 and p27^ kip1^

Total protein was extracted from AtT-20 cells and quantified using the BCA protein assay kit (Thermo Scientific, Massachusetts). Whole-cell protein extracts were resolved by SDS-PAGE and transferred to a polyvinylidene membrane (Bio-Rad, USA) using an electroblotter (Bio-Rad). Membranes were blocked with 5.0% nonfat milk for 1 h at room temperature, followed by overnight incubation at 4°C with primary antibodies to Skp2 and p27^kip1^ at a dilution of 1∶2000 (Cell Signaling, Massachusetts, USA) and 1∶2000 (BD, Biosciences, New Jersey USA), respectively. Primary antibody binding was detected using a HRP-conjugated secondary antibody (anti-rabbit, 1∶2000, SouthernBiotech, San Diego, USA; anti-mouse, 1∶3000, Amersham Biosciences, Buckinghamshire, United Kingdom) and Immunobilon Western Chemiluminescent HRP substrate (Millipore, Massachusetts, USA). Internal control was performed by incubating the membranes with beta-actin antibody 1∶2000 (Sigma, St. Louis, USA) followed by incubation with peroxidase-conjugated antibody, and the reaction was developed as described above. Densitometric analysis of bands was performed using the software Image J (http://rsb.info.nih.gov/ij/).

### Statistical Analysis

Data for MTT, clonogenicity, cell cycle, apoptosis, ACTH determination, connexin levels and densitometric analysis were expressed as mean±standard deviation of three independent experiments. Data obtained from the iontophoretic assays were expressed as mean±standard deviation of two independent experiments. Statistical comparisons were carried out by one-way ANOVA followed by Tukey’s test for cell viability analyses, and one-way ANOVA followed by Bonferroni’s test for other analyses. All statistical analyses were performed with the GraphPad Prism 4.0 and Statistics 6.0 programs. The differences between the treated and non-treated groups were considered significant when *p*<0.05.

## Results

### Beta-carotene and Lycopene Inhibit Cell Viability of AtT-20 Cells

**Figure 1:**
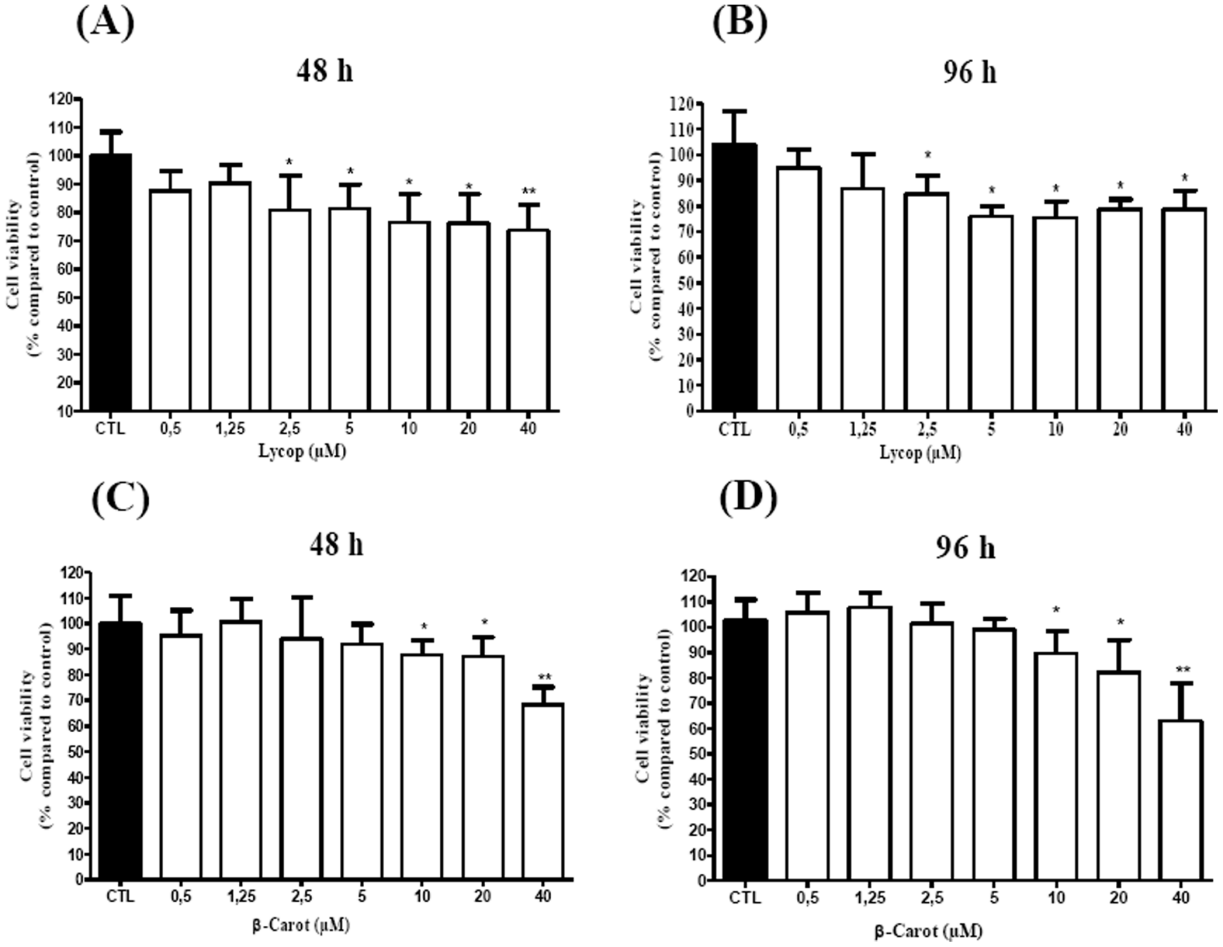
Cell viability of AtT 20 cells treated with lycopene or beta-carotene. The cells were treated with lycopene (A and B) (0.5–40 µM) or beta-carotene (C and D) (0.5–40 µM) for 48 and 96 h, and the MTT assay was done. The results are expressed as % compared to the control, and expressed as mean±standard deviation of 3 independent experiments, each performed with at least 3 replicates. *indicates significant differences from the control group (*p<0.05, **p<0.01).

AtT-20 cells were treated with lycopene or beta-carotene at physiological and supra-physiological concentrations, ranging from 0.5 to 40 µM. The treatment of lycopene for 48 and 96 h induced an inhibition of cell viability from the concentration of 2.5 µM (by 25% compared with the control group, *p*<0.05), and the maximum inhibition was obtained with 40 µM (30%, *p*<0.01) **(** **Figures 1A and 1B** **)**. On the other hand, the treatment with beta-carotene for 48 and 96 h decreased cell viability from the concentration of 10 µM (around 20%, *p*<0.05), and the maximum inhibition was observed with 40 µM **(** **Figures 1C and 1D** **)**.

### Beta-carotene and Lycopene Inhibit the Clonogenic Ability of AtT-20 Cells

**Figure 2:**
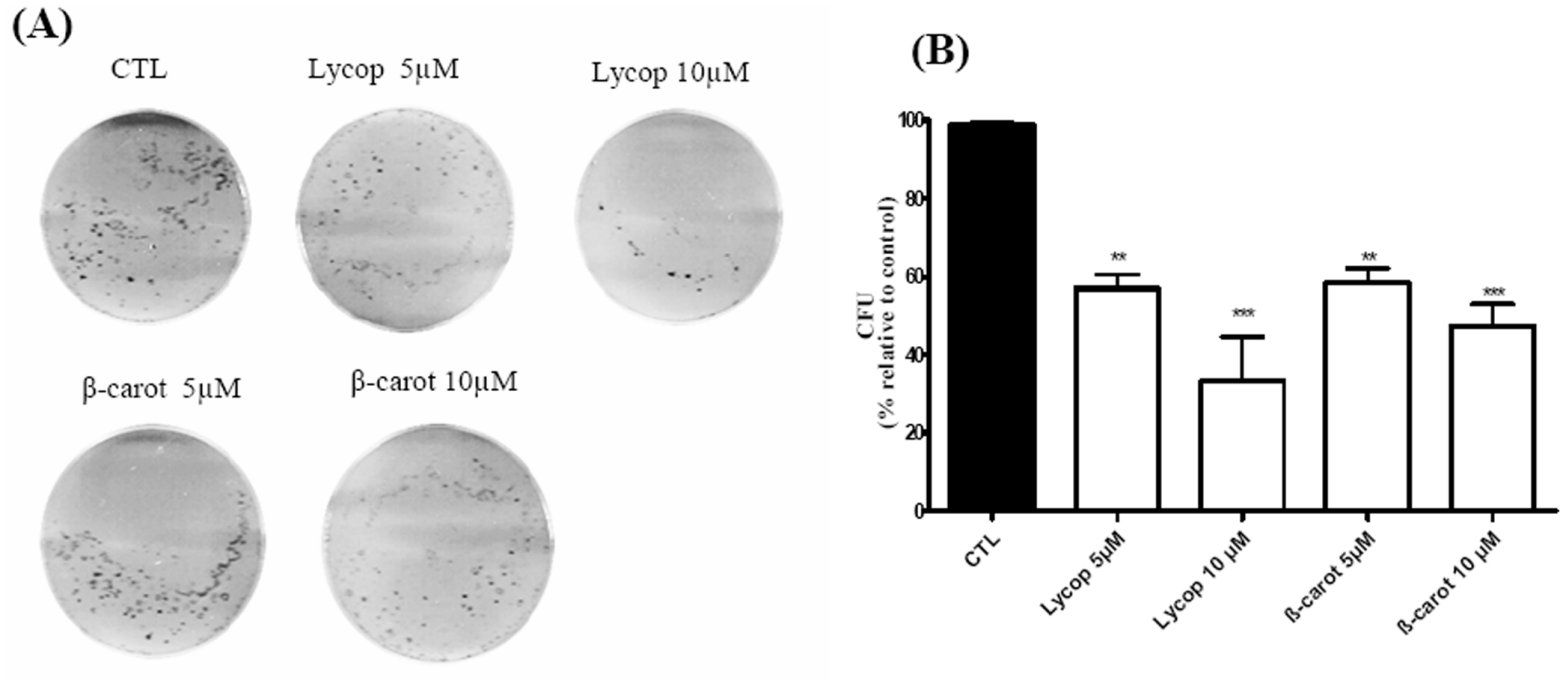
Formation of AtT-20 colonies. The number of AtT-20 colonies was determined after 21 days of culture in DMEM supplemented with 10% FCS containing lycopene (Lycop) and beta-carotene (beta-carot) at concentrations of 5 and 10 µM. The number of colonies formed was detected by crystal violet staining. Phase contrast microscopy of AtT-20 cell colonies was observed on 6-well culture plates (**A**) and quantitative representation of the colonies formed (**B**). Data are presented as mean±standard deviation of 3 independent experiments, each performed at least in duplicate. *indicates significant differences from control group (**p<0.01, ***p<0.001).

The next step was to analyze the effect of lycopene and beta-carotene on the clonogenic property of AtT-20 cells. AtT-20 cells were plated in 6-well plates at a density of 10^3^ cells/well with complete growth medium. The medium was then replaced to complete growth medium containing lycopene or beta-carotene, at the concentrations of 5 and 10 µM, and the ability of AtT-20 to form colonies was monitored over the following 21 days. According to the literature, cell groups with fewer than 50 cells were not considered as colonies [21]. Our data showed that the clonogenic ability of AtT-20 cells was inhibited in the presence of both lycopene and beta-carotene (5 and 10 µM) **(** **Figure 2A** **)**. Maximum reduction of clonogenic ability was obtained when 10 µM lycopene (about 70%, ***p<0.001) and 10 µM beta-carotene (about 50%, ***p<0.001) were used **(** **Figure 2B** **)**.

### Beta-carotene and Lycopene Modulate the Cell Cycle of AtT-20 Cells

**Figure 3:**
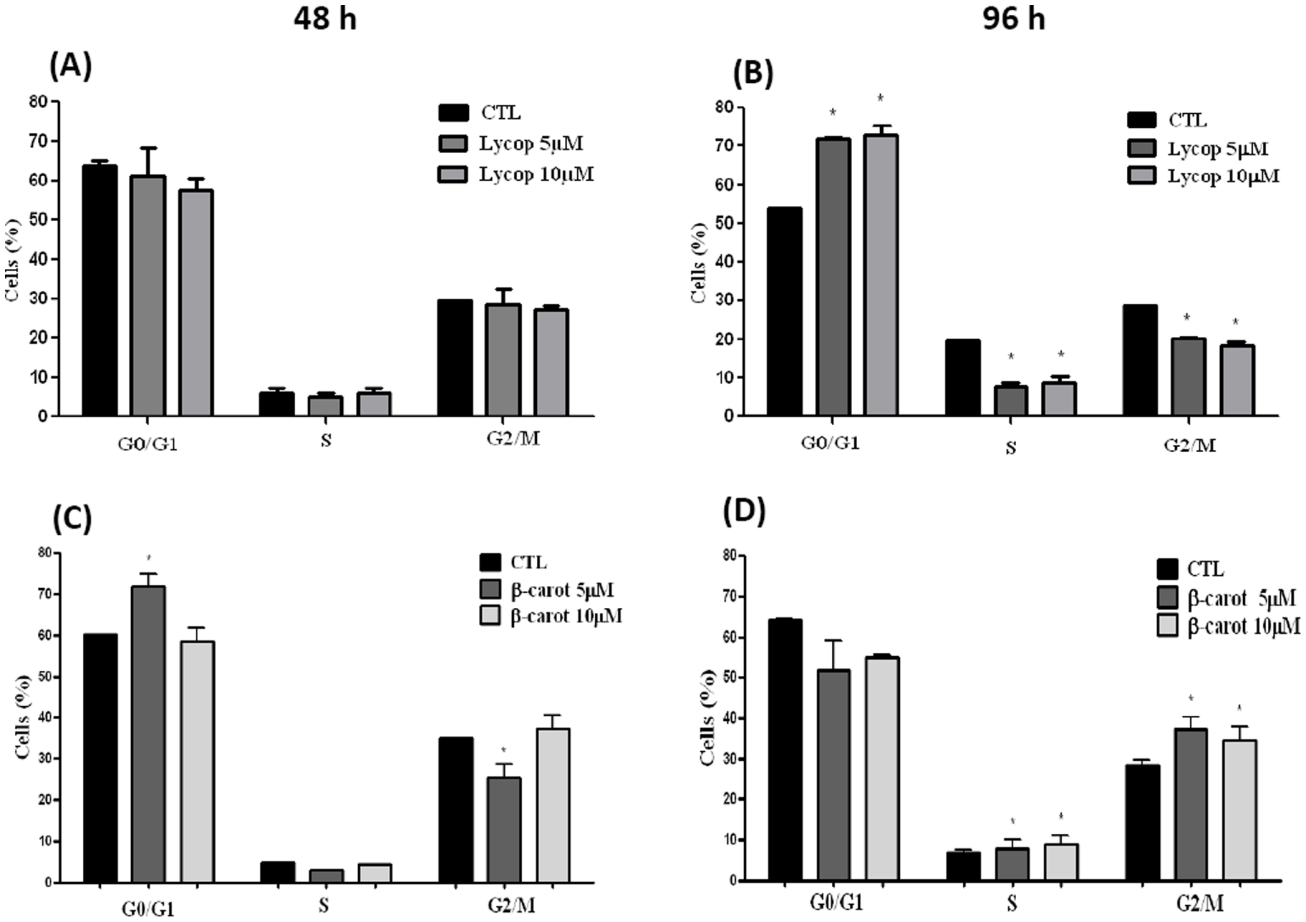
Analyses of AtT-20 cell cycle by flow cytometry under lycopene (Lycop) or beta-carotene (beta-carot) stimulation. The cells were treated with lycopene or beta-carotene at concentrations of 5 and 10 µM for 48 and 96 h, washed, and labeled with Vindeleov solution. Then, the cell cycle phases were determined using flow cytometry. The results are expressed as % of cells in G0/G1, S and G2/M phases. No significant differences between cell cycle phases were observed when AtT-20 cells were treated with 5 and 10 µM lycopene for 48 h (**A**), but after 96 h (**B**), cells in both concentrations showed an increase in G0/G1 and a decrease in S and G2/M phases. For the cells treated with beta-carotene for 48 h (**C**), the % of cells in G0/G1 and S phases increased at the concentration of 5 µM; whereas at 96 h (**D**) the % of cells in S and G2/M phases increased. Data are expressed as mean±standard deviation of 3 independent experiments, each performed with at least 4 replicates. *indicates significant differences from control group (*p<0.05).

We next questioned whether lycopene and beta-carotene would have any effect on cell cycle arrest. To address this, we performed a flow cytometry assay. After 48 h of treatment with lycopene (5–10 µM), no changes in cell cycle profile of AtT-20 cells were detected when compared to untreated cells **(** **Figure 3A** **)**. However, after 96 h of treatment, lycopene caused an increase in the percentage of cells in the G0/G1 phase, with a corresponding decrease in the S and G2/M phases, indicating a growth arrest of AtT-20 cells after that time **(** **Figure 3B** **)**. Beta-carotene caused a change in the cell cycle that differed from the effects observed with lycopene. After 48 h, the lowest concentration of beta-carotene (5 µM) increased the percentage of cells in the G0/G1 phase and decreased the amount of cells in the G2/M phase **(** **Figure 3C** **)**. However, after a longer period of treatment with beta-carotene, the proportion of cells in the G2/M and S phases increased, irrespective of the concentration used **(** **Figure 3D** **)**.

### Beta-carotene and Lycopene Induce Apoptosis in AtT-20 Cells

**Figure 4:**
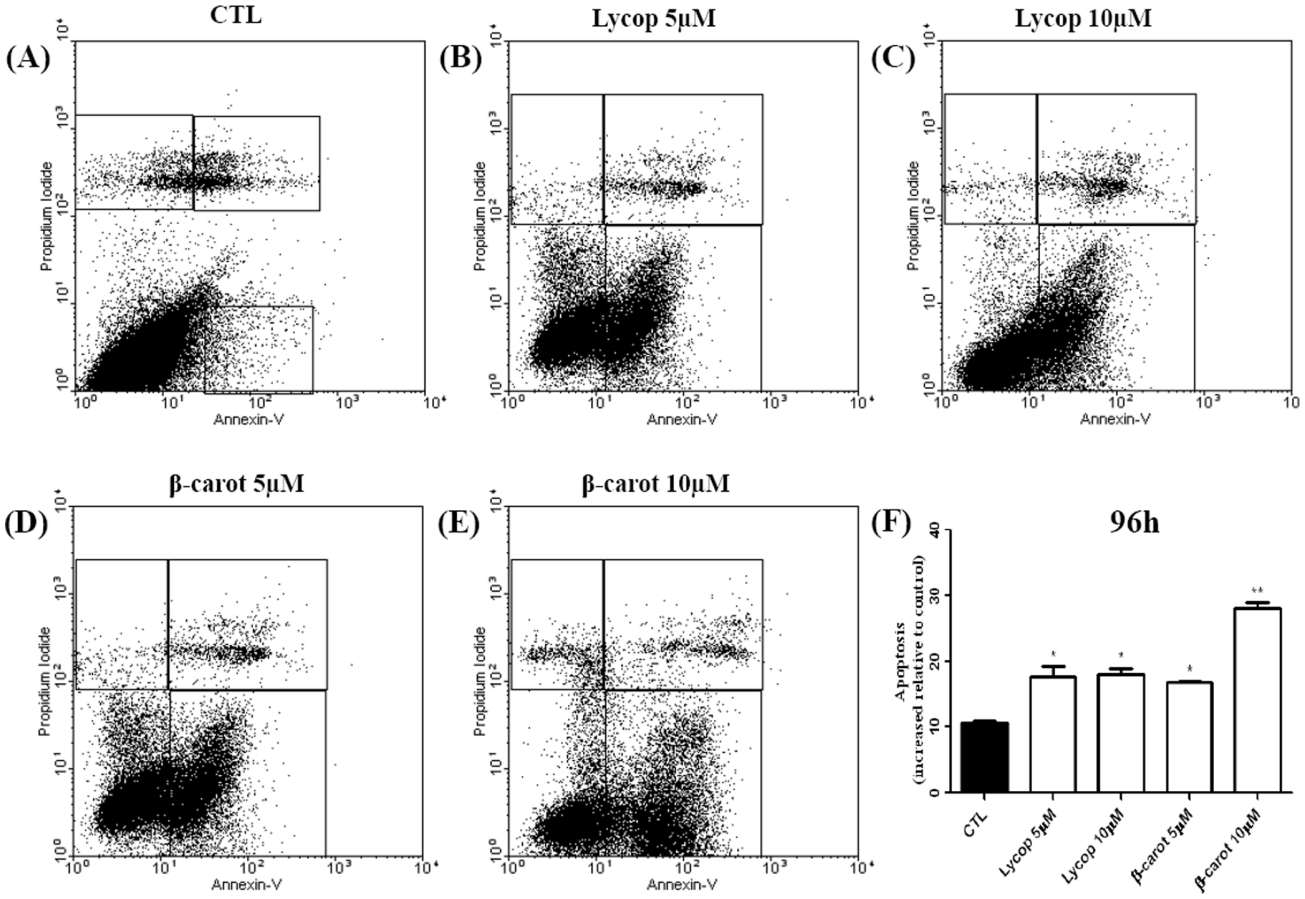
Detection of apoptotic AtT-20 cells by flow cytometry under lycopene or beta-carotene stimulation at the concentrations of 5 and 10 µM for 96 h. When the cells were treated with lycopene and carotene, the apoptosis rate increased significantly at concentrations of 5 and 10 µM. Beta-carotene at 10 µM induced a greater increase in the rate of apoptosis compared with the other experimental conditions. Data are expressed as mean±standard deviation relative to the control, of 3 independent experiments, each performed with at least 3 replicates. *indicates significant differences from control group (*p<0.05, **p<0.01, ***p<0.001).

Flow cytometry analysis showed that treatment for 48 h with either lycopene or beta-carotene at concentrations of 5 and 10 µM did not induce apoptosis (data not shown). However, when cells were treated under the same conditions for 96 h, an increase in the number of apoptotic cells was detected **(** **Figure 4** **)**.

### Lycopene and Beta-carotene Decrease ACTH Secretion

**Figure 5:**
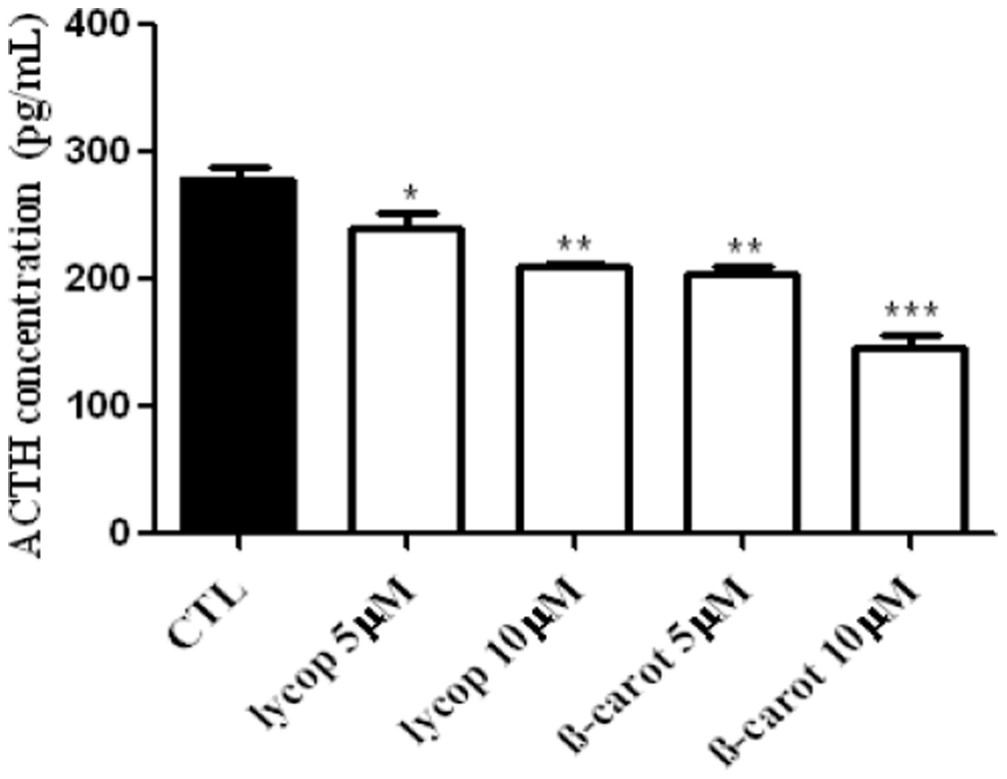
ACTH secretion by AtT-20 cells. Cells were treated with 5 and 10 µM of beta-carotene or lycopene for a period of 24 h, and ACTH levels were determined in the supernatant by immunoassay. The treatment reduced the ACTH secretion in a dose-dependent manner. Data are expressed as mean±standard deviation pg/mL, of 3 independent experiments, each performed with at least 2 replicates. *indicates significant differences from control group. (*p<0.05, **p<0.01, ***p<0.001).

ACTH secretion by AtT-20 cells decreased after lycopene or beta-carotene (5 and 10 µM) treatment for 24 h **(** **Figure 5** **),** in a concentration-dependent manner.

### Beta-carotene and Lycopene Modulate the Levels of Total Cx43 and Phosphorylated-Cx43

**Figure 6:**
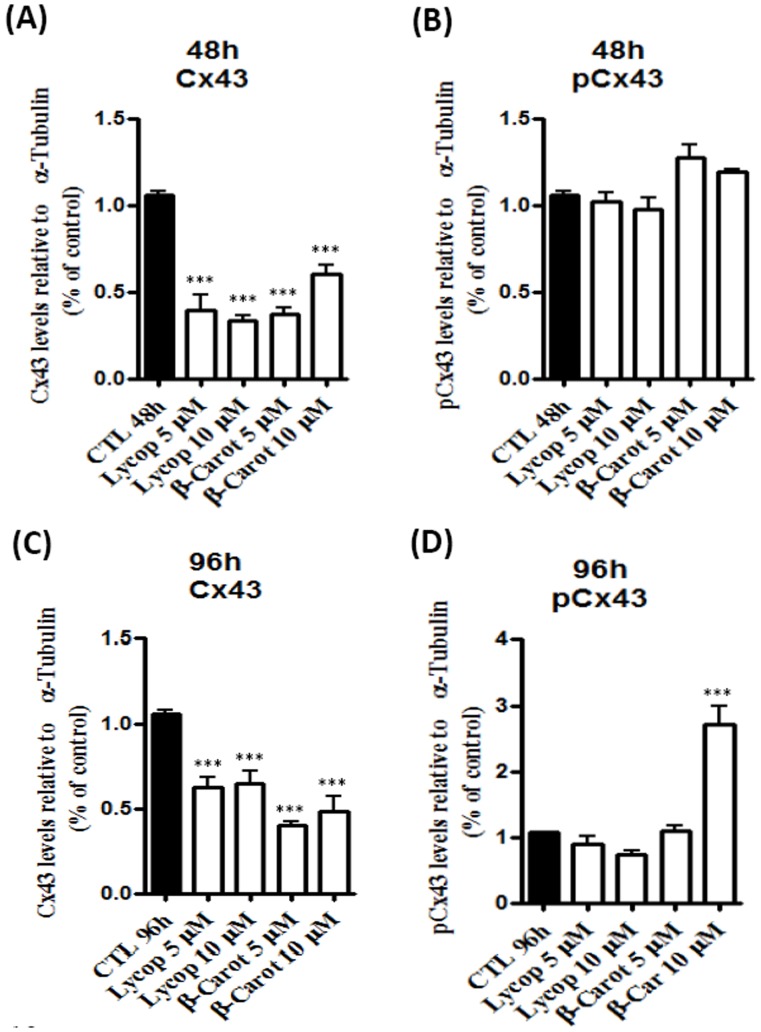
Levels of total connexin 43 (Cx43) and phosphorylated connexin 43 (pCx43) were determined by enzyme-linked immunosorbent. AtT-20 cells were treated with lycopene (Licop) or beta-carotene (β-car) at concentrations of 5 and 10 µM for 48 and 96 h. Cx 43 levels decreased under carotenoid treatment at 48 h (**A**) and 96 h (**C**). However, the levels of pCx43 increased only after 96 h under 10 µM beta-carotene stimulation (**B** and **D**). Data are expressed as mean±standard deviation of 3 independent experiments, each performed with at least 2 replicates. *indicates significant differences from control group. (***p<0.001).

We observed that AtT-20 cells treated with beta-carotene or lycopene for 48 and 96 h showed significant reductions of total connexin 43 levels **(** **Figures 6A and 6C** **)**. However, the expression of phosphorylated-Cx43 was significantly increased after 96 h of treatment only with beta-carotene at the concentration of 10 µM (**Figures 6D** **)**.

### Beta-carotene and Lycopene Block the Intercellular Communication via Gap Junction

**Figure 7:**
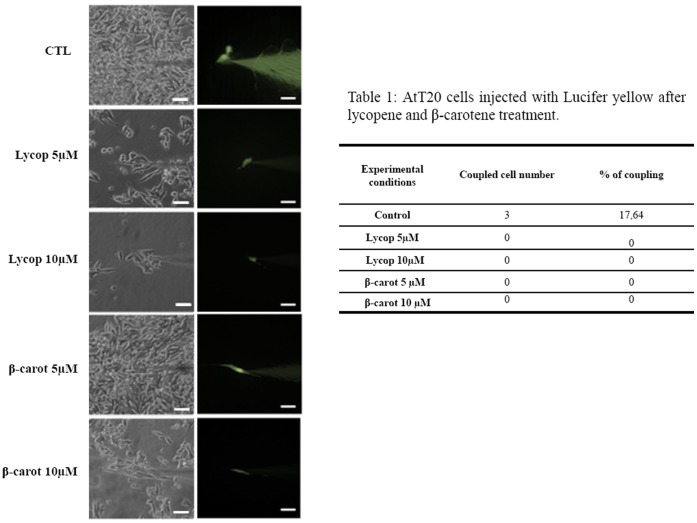
Photomicrographs from bright- and dark-field phase-contrast microscopy of AtT-20 cells treated with lycopene or beta-carotene 5 and 10 µM for 48 h and injected with Lucifer yellow iontophoretic dye (A). A transfer of the dye occurred only in the control condition; the treatments completely blocked the dye transfer between neighboring cells. In **B**, the number of coupled cells and the rate of cell coupling (%). Phase-contrast images were taken from random fields. Bar: 80 µm.

AtT-20 cells cultured under beta-carotene or lycopene stimulation (5 and 10 µM) for 48 h were injected using Lucifer Yellow dye solution. The profile of intercellular coupling is shown in **Figure 7A**. Non-treated AtT-20 cells showed a coupling rate of approximately 17.6%; however, no coupling cells were observed after the carotenoid treatments **(** **Figure 7B** **)**, which strongly suggests that the effects of the carotenoids might be mediated by independent connexin gap junction formation.

### Skp2 and p27^kip1^ Protein Levels

**Figure 8:**
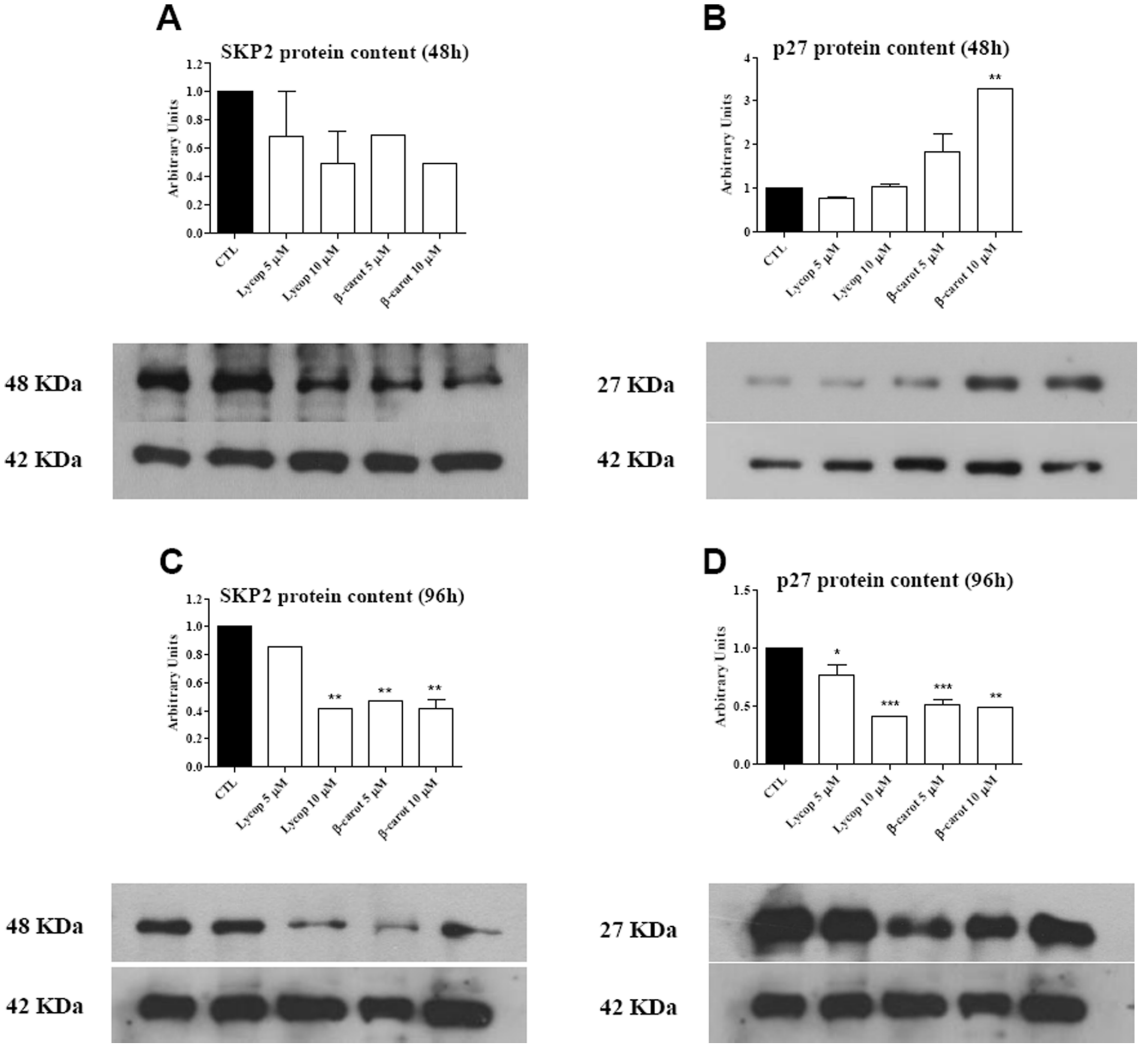
Western blot analysis of Skp2 and p27 protein levels in AtT-20 cells under lycopene (Lycop) or beta-carotene (β-carot) stimulation. Treatment with Lycop or β-carot at 5 and 10 µM for 48 h did not change the Skp2 levels significantly, although a trend decrease was observed (**A**); after 96 h, Skp2 levels in Lycop 10 µM were decreased (**C**). When the cells were treated with carotenoids at 5 and 10 µM for 48 h, p27 protein levels increased significantly in β-carot 10 µM (**B**); after 96 h of treatment, both concentrations of carotenoids decreased p27 protein levels (**D**). (*p<0.05, **p<0.01, ***p<0.001).

The role of Skp2 as an oncogene responsible for down regulation of p27^kip1^ protein levels is well established in a wide variety of tumors, including pituitary tumors. AtT-20 cells treated with lycopene or beta-carotene (5 and 10 µM) for 48 h showed a decreasing tendency of Skp2 protein levels (**Figure 8A** **)**. p27^Kip1^ protein levels were increased by beta-carotene treatment (5 and 10 µM) for 48 h, although no effects were seen in lycopene treated cells (**Figure 8B**). The cells under lycopene 10 µM and beta-carotene (5 and 10 µM) treatment for 96 h significantly decreased Skp2 expression **(** **Figure 8C** **)**. Finally, the cells treated with lycopene or beta-carotene (5 and 10 µM) for 96 h showed a significant decrease in p27^kip1^ levels **(** **Figure 8D** **)**.

## Discussion

The use of some inhibitors of ACTH production has been described, but they are ineffective in treating Cushing’s disease [22]. However, it has recently been reported that the use of pasireotide, a somatostatin analogue, has shown success in treating this disease [23], [24]. At the same way, it is essential to prospect for new drugs that could constitute an efficient pharmacological approach to treat Cushing’s disease.

Lycopene and beta-carotene are potent antioxidant agents, and are anti-tumorigenic in different tumor cell types [11], [25]–[30]. It was demonstrated that concentrations with less than 20 µmol/L of beta-carotene and lycopene were sufficient to significantly reduce the viability of human prostate tumor cells PC-3 and DU 145 [25]. Recently, it was also demonstrated that lycopene and beta-carotene impair the growth of prostate tumor cells, which was associated with a decrease in proliferating cell nuclear antigen expression and with the insulin-like growth factor I signaling pathway [11]. Also in HT-29 colon adenocarcinoma cells, it was reported that beta-carotene-rich tomato lycopene beta-cyclase inhibited cell growth [12].

In the present study, we demonstrate for the first time that lycopene and beta-carotene show anti-tumorigenic effects on ACTH-secreting pituitary adenoma AtT-20 cells. These carotenoids inhibit AtT-20 cell proliferation and colony formation, induce apoptosis and cell cycle arrest, and reduce ACTH secretion. Lycopene and beta-carotene were used in a range of physiological and supra-physiological concentrations, from 0.5 to 40 µM [31], [32], [33]. After 48 and 96 h of treatment, both carotenoids caused a maximum decrease in AtT-20 cell viability at the concentration of 40 µM.

Previous reports have demonstrated the effects of natural products on pituitary tumor cells. Using rat lactotroph cell lines, GH3 and MMQ cells, Miller et al. [33] at the first time report that curcumin had an important and significant effect on GH3 and MMQ cell proliferation, apoptosis, hormone secretion and colony formation ability of pituitary tumor cells. The growth-inhibitory effect of curcumin was accompanied by decreased expression of cyclin D3 and ser 780 phosphorylation of retinoblastoma protein. Finally, they showed that low concentrations of curcumin enhanced the growth-inhibitory effect of bromocriptine on MMQ cell proliferation. Besides, Schaaf et al. [3] demonstrated that curcumin acts as an anti-tumorigenic and hormone secretion suppressive agent in murine and human pituitary cells *in vitro* and *in vivo*. Schaaf et al. [34] also observed that curcumin affects growth, the apoptotic index and functions in folliculostellate cells, a non-endocrine cell. These effects could have consequences for pituitary homeostasis as well as tumor pathogenesis. Recently, Bangaru et al. [4] demonstrated that curcumin was able to inhibit the proliferation and colony formation of ACTH-secreting pituitary adenoma cells in a concentration-dependent manner (2.5–200 µM). Here, we observed inhibition of AtT-20 colony formation at carotenoid concentrations of 5 and 10 µM, similar to results observed in other studies which used natural compounds against pituitary tumor cells [3], [4], [33]. Thus, we suggest that lycopene and beta-carotene might be less potent than curcumin in inhibiting the clonogenic ability of AtT-20 cells.

Several studies have shown that carotenoids can induce cell cycle arrest and apoptosis. Arrest of tumor growth is often associated with induction of cell apoptosis, and carotenoids have been shown to induce apoptosis in tumor cells, in parallel with growth inhibition. In breast cancer cells treated with 2–3 µM lycopene, there was a progressive inhibition of the G1 phase cell cycle [35]. In a colon adenocarcinoma cell line, lycopene and beta-carotene inhibited the cell cycle progression in the G0/G1 and G2/M phases, and increased apoptotic cells by 15% [12]. Our results reveal important differences between lycopene and beta-carotene with respect to cell cycle arrest. Lycopene (5 and 10 µM) caused an increase of cells in the G0/G1 phase only after 96 h, whereas beta-carotene (5 µM), after a shorter time (48 h), increased the cells in the same G0/G1 phase, leading to increased apoptosis. After 96 h of beta-carotene (5 and 10 µM) treatment, it was observed an increase the cells in G2/M phase. These data can be explained by the difference in the polarity of the carotenoid molecule, because the cellular uptake of lycopene is delayed compared to beta-carotene, as demonstrated in murine hepatic stellate cells [36], [37]. Although we did not find studies of carotenoids effects on tumoral pituitary cells in the literature, previous studies in breast and colon cancer models showed, similar to our results, that lycopene promoted cell cycle modification and increased cells in the G0/G1 phase [38]–[40]]. In contrast, Hwang and Bowen [41] showed an interference of lycopene with the cell cycle of LNCaP cells in the G2/M phase. It was also reported that beta-carotene inhibit the growth of several human colon adenocarcinoma cell lines by inducing cell cycle arrest in G2/M phase and apoptosis [42]. In addition, previous studies reported that carotenoids effects on cell cycle arrest involve a downregulation of cyclins, including cyclin E and cyclin D1, and/or a upregulation of cyclin A and p27 [13], [39], [43].

Carotenoids have the ability to regulate junctional communication, which might be related to the suppression of the malignancy phenotype [44]–[48]. The well-orchestrated interaction between the intracellular and extracellular microenvironment is a prerequisite for maintenance of tissue homeostasis. The role of intercellular communication has been widely debated in the process of tumorigenesis. Intercellular communication is mainly mediated by gap junctions that are formed by adjacent hemichannels, which are composed by six subunits of connexins (Cx) [17]. These junctions control many physiological processes, including cell growth, cell differentiation, cell death and cell signaling [49], [50]. These biological events are related to connexin phosphorylation status, and constitute an important mechanism to control the gap junction intercellular communication; connexin phosphorylation status controls intercellular channel opening and closing, and consequently the signals passage between the cells [51]–[53].

Generally, tumor cells are deficient in communicating junctions [54]–[56], and intercellular communication is important for the control of normal cell growth [57]. Therefore, we evaluated the functionality of gap junctions formed by tumor cell line AtT-20; the control cells showed a rate of coupling of 17.6%, and the treatment with carotenoids (5 and 10 µM) completely blocked cell coupling. These results could suggest: 1) carotenoids can block intercellular communication via gap junction inhibiting the transference of survive and/or apoptosis signals between neighboring cells; and/or 2) since lycopene and beta-carotene induced a decrease in the total Cx43 expression and an increase in the pCx43 levels, these effects show that pCx43 is most likely to work in another signaling pathway, independently of gap junction formation. Recent studies have demonstrated the role of pCx-43 in the expression of growth regulators and cell death; and it has been suggested that connexins can directly affect gene transcription or act as modulators of proteins that control the cell cycle such as Skp2, and consequently p27^kip1^ [17]. The role of Skp2, which acts as an an oncogene through targeting p27^kip1^ for degradation, has been observed in a wide variety of tumors, including some pituitary adenomas [58]–[61]. We examined the possibility of a correlation between Skp2 and p27^kip1^ expression in ACTH-secreting pituitary tumor cells under carotenoids stimulation. Our results suggest a significant inverse correlation between Skp2 and p27^kip1^ protein levels, mainly in beta-carotene stimulation for 48 h, which might be associated with anti-proliferative and pro-apoptotic effects induced by beta-carotene treatment on pituitary tumor cells. However, the decrease in Skp2 levels in AtT-20 cells treated with lycopene (5 and 10 µM) and beta-carotene (5 µM) for 48 h did not translate to p27 upregulation. The same results were found by Huang et al. [62] using breast cancer cells as model. In addition, carcinoma prostate cells treated with lycopene (2.5–10 µM) reduced intracellular total cholesterol by decreasing enzymes related to cholesterol biosynthesis and by inactivating Ras also accompanied by an decrease in cyclin D1, phospho-Akt levels and by a increase in p21, p27 and p53 levels, and the Bax/Bcl-2 ratio [40]. Also, others mechanisms of action could be related to cell cycle arrest and apoptosis promoted by carotenoids and chemically related compounds. Giacomini et al. [63] demonstrated that the effect of retinoic acid, a product of carotenoids metabolization, has been related to BMP-4 (bone morphogenetic protein-4), a member of transforming growth factor superfamily. It was observed that retinoic acid induced both BMP-4 transcription and expression, and its antiproliferative effect is blocked in Smad-4 and Noggin-transfected AtT-20 cells that not respond to BMP-4. Using a transgenic zebra fish, Liu et al. [64] (2011) demonstrated that R-roscovitine, a CKD2/cyclin E inhibitor, was able to suppress ACTH expression, promotes corticotroph tumor cell senescence and cell cycle exit by up-regulating p27, p21 and p57, and downregulating cyclin E expression. Taken together, it seems that carotenoids effects on proliferation and apoptosis are related to p27 expression decreasing and Skp2 increasing. However, other studies will be necessary to clarify the exact molecular mechanism which lycopene and beta-carotene act.

High levels of ACTH and hypercortisolism are associated with the progression of Cushing's disease. Suppression or at least normalization of hormone secretion is the major goal in endocrine-active microadenomas. In our study, it was observed that ACTH secretion decreased in AtT-20 cells treated with carotenoids. However, the effect of carotenoids on the production of anterior pituitary hormones is still unknown. We do know that drugs used to treat Cushing’s disease as somatostatin analogues and dopamine agonist are able to decreased hormone secretion by different mechanisms. The hormonal blockade when using somatostatin analogues and dopamine agonist suggests the participation of second messengers such as calcium, phospholipase C and 3-inositol triphosphate, and POMC expression regulation [65], [66]. However, the differential gene expression of POMC-related processing enzymes, transcription factors, and receptors responsible for ACTH secretion by non-pituitary and pituitary ACTH-secreting tumors remains obscure. Recently, Tani et al. [67] try to determine the gene expression profile of transcription factors (*Tpit*, *NeuroD1* and *IKZF1*), proprotein convertase (*PC*) *1/3* and *PC2*, and several key receptors linked to ACTH secretion, including corticotrophin releasing hormone receptor (*CRHR1*), vasopressin receptor 1b (*V1bR*), somatostatin receptor (*SSTR*) subtype-2, -5 and dopamine receptor type 2 (*D2R*) in non-pituitary and pituitary ACTH-secreting tumors. And then, these authors concluded that IKZF1 has a potential implication on cell differentiation and hormone secretion while PC2 enhanced processing activity of mature ACTH. On the other hand, SSTR2-5 also involved in ACTH secretion, thus its agonist could be used as a potential therapeutic in ectopic ACTH syndrome. Based on these results, we can speculate that carotenoids could be regulating the hormonal secretion pathways and/or POMC expression. However, further studies are required to elucidate how the molecular targets are affect by carotenoids in AtT-20 hormone secretion.

We report here that lycopene and beta-carotene, apart from inhibiting cell proliferation, also potently suppress ACTH secretion. Lycopene and beta-carotene are able to produce these effects by a mechanism that probably involves the regulation of connexin 43, Skp2 and p27^kip1^ expression, suggesting that these compounds might correspond to novel pharmacological approaches for the treatment of pituitary adenomas.
