# Correction: Lycopene and Beta-Carotene Induce Growth Inhibition and Proapoptotic Effects on ACTH-Secreting Pituitary Adenoma Cells

**DOI:** 10.1371/journal.pone.0149157

**Published:** 2016-02-05

**Authors:** Natália F. Haddad, Anderson J. Teodoro, Felipe Leite de Oliveira, Nathália Soares, Rômulo Medina de Mattos, Fábio Hecht, Rômulo Sperduto Dezonne, Leandro Vairo, Regina Coeli dos Santos Goldenberg, Flávia Carvalho Alcântara Gomes, Denise Pires de Carvalho, Mônica R. Gadelha, Luiz Eurico Nasciutti, Leandro Miranda-Alves

The authors would like to correct Fig 2A and Fig 4. In Fig 2A, while preparing the figure for publication, the same image was erroneously used for the Lycop 5 μM plate and the beta-carot 10μM plate. The authors have provided a corrected version of Fig 2, which includes the correct image for the Lycop 5 μM plate. In Fig 4, while preparing the figure for publication, the same image was erroneously used for the Lycop 5μM flow cytometry plot and the beta-carot 5μM plot. The authors have provided a corrected version of Fig 4, which includes the correct image for the beta-carot 5μM plot. The underlying data and unedited images for Fig 2A and Fig 4 are available as Supporting Information. The authors confirm that these errors do not alter their results.

**Fig 2:**
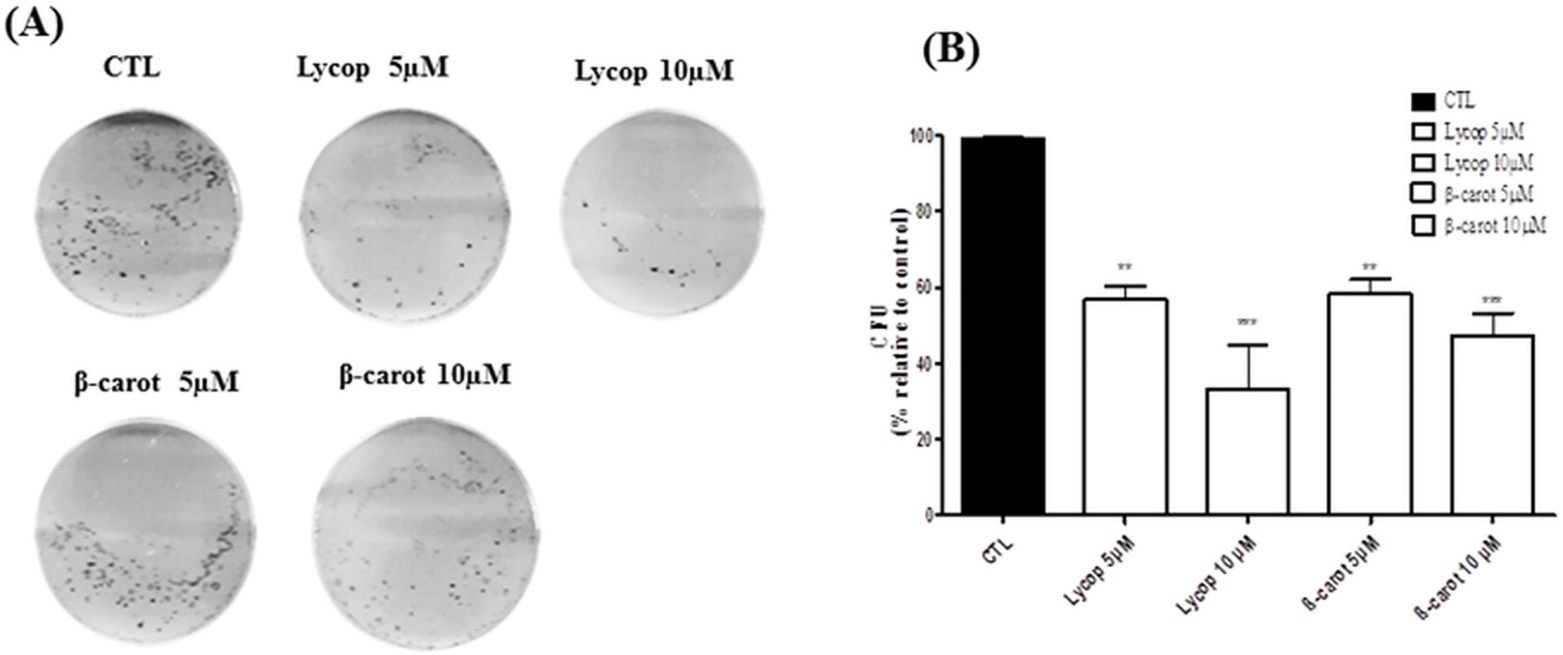
Formation of AtT-20 colonies. The number of AtT-20 colonies was determined after 21 days of culture in DMEM supplemented with 10% FCS containing lycopene (Lycop) and beta-carotene (beta-carot) at concentrations of 5 and 10 μM. The number of colonies formed was detected by crystal violet staining. Phase contrast microscopy of AtT-20 cell colonies was observed on 6-well culture plates (A) and quantitative representation of the colonies formed (B). Data are presented as mean±standard deviation of 3 independent experiments, each performed at least in duplicate. *indicates significant differences from control group (**p<0.01, ***p<0.001).

**Fig 4:**
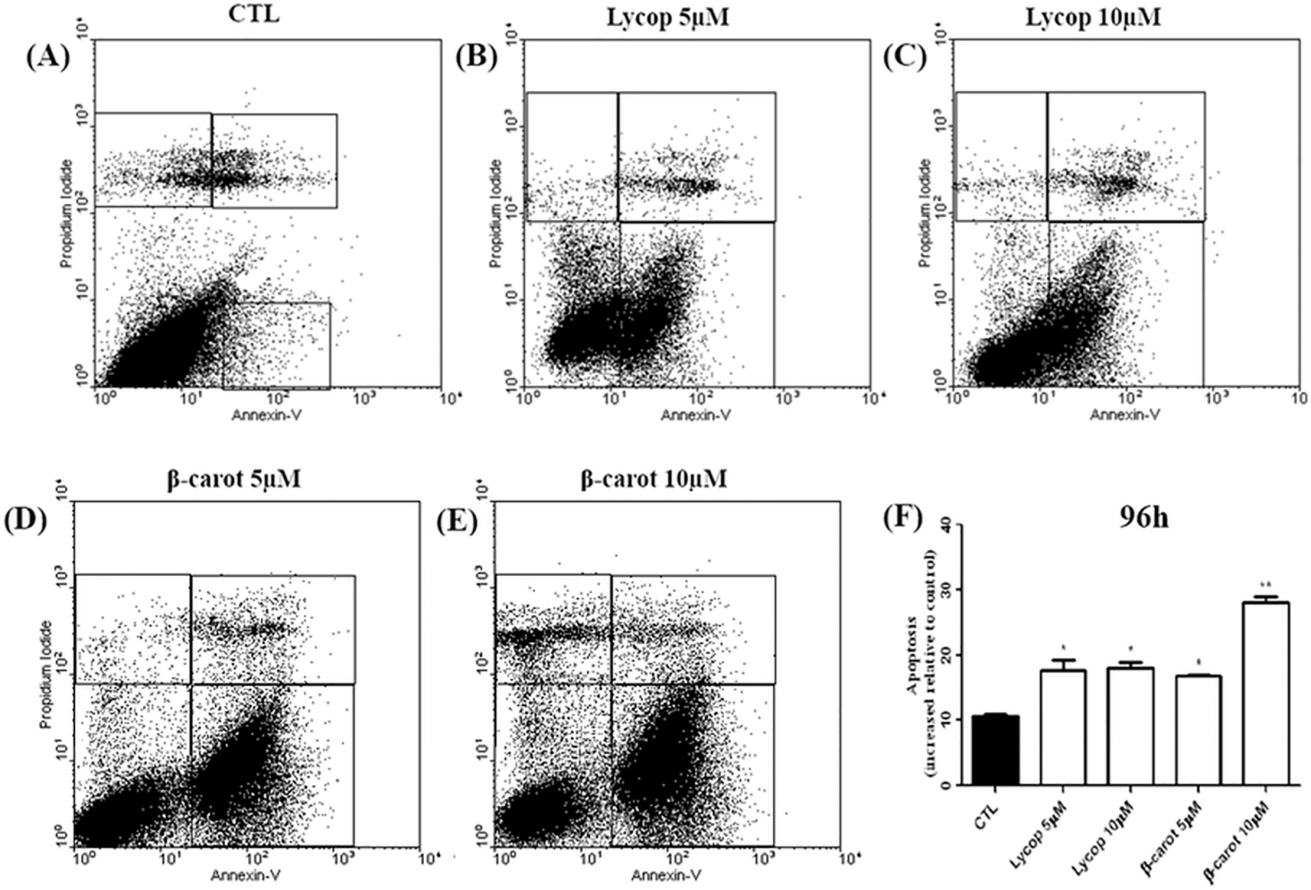
Detection of apoptotic AtT-20 cells by flow cytometry under lycopene or beta-carotene stimulation at the concentrations of 5 and 10 μM for 96 h. When the cells were treated with lycopene and carotene, the apoptosis rate increased significantly at concentrations of 5 and 10 μM. Beta-carotene at 10 μM induced a greater increase in the rate of apoptosis compared with the other experimental conditions. Data are expressed as mean±standard deviation relative to the control, of 3 independent experiments, each performed with at least 3 replicates. *indicates significant differences from control group (*p<0.05, **p<0.01, ***p<0.001).

## Supporting Information

S1 Data and Images The underlying data and unedited images for Fig 2A and Fig 4. (ZIP)
