# Supplementary material for: Correction: Lycopene and Beta-Carotene Induce Growth Inhibition and Proapoptotic Effects on ACTH-Secreting Pituitary Adenoma Cells
Source: PLoS One. 2016 Feb 5;11(2):e0149157. doi: 10.1371/journal.pone.0149157 (PMC4743989; doi:10.1371/journal.pone.0149157)
Supplement: S1 Data and Images — (ZIP) [file pone.0149157.s001.zip › RAW images (Figure 2A CFU).pptx]

## Slide 1
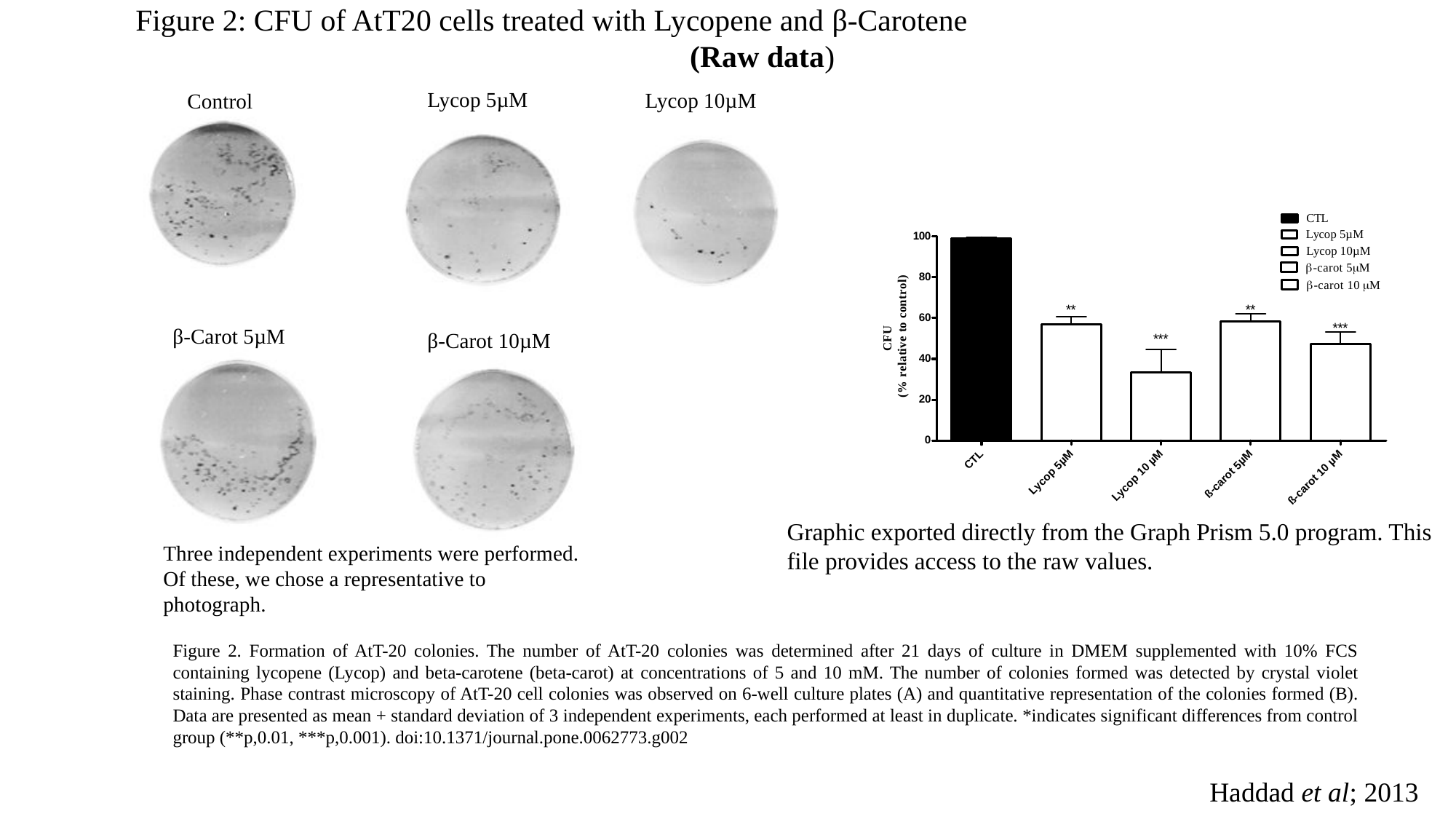

Figure 2: CFU of AtT20 cells treated with Lycopene and β-Carotene
(Raw data)
Lycop 5µM
Lycop 10µM
Control
β-Carot 5µM
β-Carot 10µM
Graphic exported directly from the Graph Prism 5.0 program. This file provides access to the raw values.
Three independent experiments were performed.
Of these, we chose a representative to photograph.
Figure 2. Formation of AtT-20 colonies. The number of AtT-20 colonies was determined after 21 days of culture in DMEM supplemented with 10% FCS containing lycopene (Lycop) and beta-carotene (beta-carot) at concentrations of 5 and 10 mM. The number of colonies formed was detected by crystal violet staining. Phase contrast microscopy of AtT-20 cell colonies was observed on 6-well culture plates (A) and quantitative representation of the colonies formed (B). Data are presented as mean + standard deviation of 3 independent experiments, each performed at least in duplicate. *indicates significant differences from control group (**p,0.01, ***p,0.001). doi:10.1371/journal.pone.0062773.g002
Haddad et al; 2013
