# Supplementary figures and images for: Correction: Lycopene and Beta-Carotene Induce Growth Inhibition and Proapoptotic Effects on ACTH-Secreting Pituitary Adenoma Cells
Source: PLoS One. 2016 Feb 5;11(2):e0149157. doi: 10.1371/journal.pone.0149157 (PMC4743989; doi:10.1371/journal.pone.0149157)

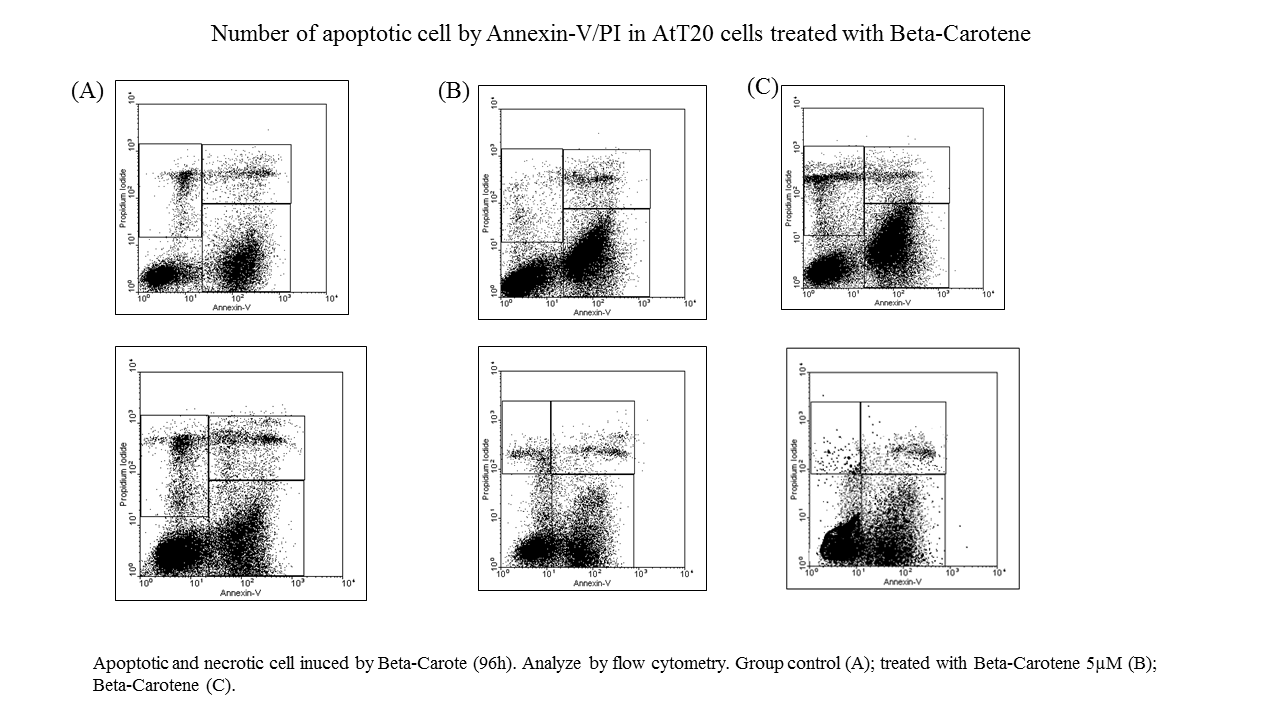

Supplement: S1 Data and Images — (ZIP) [file pone.0149157.s001.zip › Raw Figure 4 Beta-Carotene.tif]

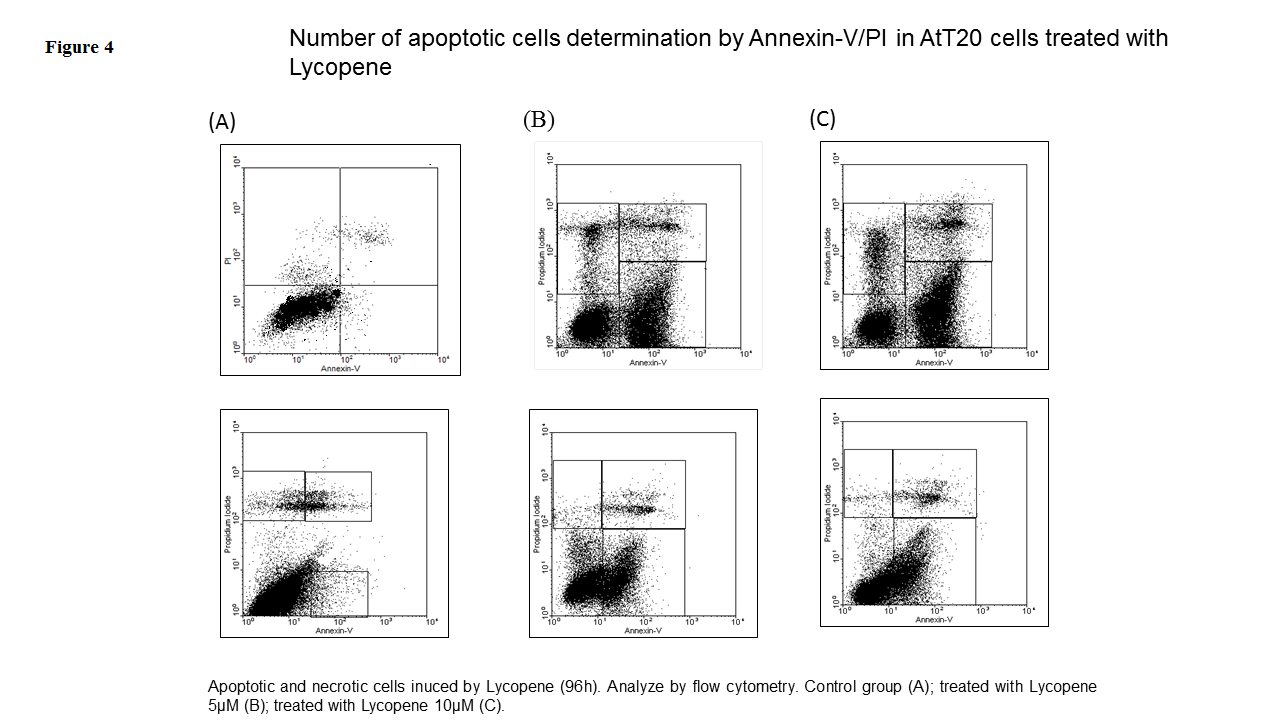

Supplement: S1 Data and Images — (ZIP) [file pone.0149157.s001.zip › Raw Figure 4 Lycopene.TIF]
